# Translucency and Color Stability of a Simplified Shade Nanohybrid Composite after Ultrasonic Scaling and Air-Powder Polishing

**DOI:** 10.3390/nano12244465

**Published:** 2022-12-15

**Authors:** Ksenia Babina, Maria Polyakova, Inna Sokhova, Vladlena Doroshina, Alexandr Zaytsev, Elena E. Nikonova, Gleb S. Budylin, Evgeny A. Shirshin, Christian Tantardini, Nina Novozhilova

**Affiliations:** 1 Department of Therapeutic Dentistry, I.M. Sechenov First Moscow State Medical University (Sechenov University), 119991 Moscow, Russia; 2 Institute of Linguistics and Intercultural Communication, I.M. Sechenov First Moscow State Medical University (Sechenov University), 119991 Moscow, Russia; 3 Laboratory of Clinical Biophotonics, Biomedical Science and Technology Park, I.M. Sechenov First Moscow State Medical University (Sechenov University), 119991 Moscow, Russia; 4 Faculty of Physics, M.V. Lomonosov Moscow State University, 119991 Moscow, Russia; 5 Institute of Spectroscopy of the Russian Academy of Sciences, 108840 Moscow, Russia; 6 World-Class Research Center “Digital Biodesign and Personalized Healthcare”, I.M. Sechenov First Moscow State Medical University (Sechenov University), 119991 Moscow, Russia; 7 Hylleraas Center, Department of Chemistry, UiT The Arctic University of Norway, 9037 Tromso, Norway; 8 Institute of Solid State Chemistry and Mechanochemistry SB RAS, 630128 Novosibirsk, Russia

**Keywords:** translucency, color stability, ultrasonic scaling, air-powder polishing, simplified shade nanocomposite resins

## Abstract

We aimed to assess the influence of professional dental prophylaxis on the translucency and color stability of a novel simplified shade nanohybrid composite material. Sixty composite disks (5 mm in diameter and 2 mm thick) of light (*n* = 30) and dark (*n* = 30) shades were prepared. The specimens were randomly divided into the following three groups (*n* = 10) according to the prophylaxis procedure used: ultrasonic scaling, air-powder polishing with sodium bicarbonate, and controls. The specimens were submitted to translucency and color analysis based on the CIELab system. Two measurements were performed before and after 48-h storage in coffee. Translucency values of untreated light and dark specimens were 9.15 ± 0.38 and 5.28 ± 1.10, respectively. Air-powder polishing decreased the translucency of the light composite specimens. Storage in coffee resulted in color changes (∆E) ranging between 2.69 and 12.05 and a mean translucency decrease ranging between −0.88 and −6.91. The samples in the light group tended to exhibit greater staining; the treatment method had no effect on ∆E. It can be concluded that light-shade composite restorations are more prone to translucency and color changes resulting from air-powder polishing and contact with staining media. However, further research using other composites and powders is required.

## 1. Introduction

Today, esthetic requirements for direct restorations are very high among dentists and patients. The world market offers a wide variety of materials for esthetic restoration. With the development of nanotechnology, dental nanocomposites have become the most commonly used restorative materials due to their high esthetic characteristics, adequate mechanical properties and resistance to abrasion, and acceptable price compared with ceramic materials [1,2,3]. To mimic tooth structure, i.e., to imitate the color, morphology, and optical properties of natural teeth, the manufacturers offer a large number of composite shades and materials with various translucency/opacity levels [4,5]. However, multiple-shade composite restorations are time-consuming [5,6], and sometimes the shade-matching procedure may be challenging [7,8]. Recently, simplified shade (also called universal) composites have been developed and claimed by manufacturers to blend well with surrounding hard tooth tissues, providing good esthetic result, while decreasing the chairside time and making shade selection more predictable [7,8,9].

The long-term color stability and natural translucency of resin composites are among the crucial factors that affect patient satisfaction and clinical longevity of esthetic restorations, especially in the anterior teeth [5,10,11]. Translucency is defined as the ability of a substance to pass through light. [12,13]. According to various studies, the translucency level of composite materials differs significantly not only between different shade groups (dentin, opaque, body, and enamel) but also within each shade group [14,15]. Color stability, which is the ability to resist color changes, may be affected by the material composition and environmental and technique-related factors [16]. It has been reported that absorption of pigments from foods and drinks, such as red wine, coffee, tea, and cola, can cause intrinsic and extrinsic staining of resin-based materials [17,18,19]. A change in color may also affect translucency, further altering the initial esthetics [16]. Discoloration of restored teeth over the long-term constitutes a reason for patient dissatisfaction, repeating the polishing procedure, or even restoration replacement [20,21,22]. The composition of the resin matrix, filler loading, and the composition and size of filler particles may also affect composite translucency [6,10,23]. Nanofillers provide advantages in optical properties, surface gloss, and smoothness [2,24]. They can also provide high translucency and resistance to staining over long periods of time [25].

Apart from material characteristics, translucency and color stability may be influenced by composite surface roughness [26,27]. The initial surface roughness of composite resin depends on intrinsic and extrinsic factors. Intrinsic factors include material composition [28] and the polymerization procedure [29], while extrinsic factors include the finishing technique and finishing and polishing systems applied [30,31]. In the long term, the surface roughness may be altered by patient-related factors including dietary habits [32,33] and smoking [33,34] and various dental prophylaxis procedures [35,36].

To the best of our knowledge, few studies assessed the effect of air-powder polishing on the translucency and color stability of composite resins, and no studies assessed that of ultrasonic scaling. Furthermore, with the introduction of simplified shade resin-based restorative materials, it is important to investigate the color stability and translucency of these materials in order to draw clinicians’ awareness to possible long-term esthetic outcomes.

The aim of our study was to assess the influence of professional dental prophylaxis on the translucency and color stability of a novel simplified shade composite material.

## 2. Materials and Methods

Ethical approval was not necessary as the study did not include human participants or animals.

### 2.1. Sample Preparation

We used a simplified universal nanohybrid composite (OptiShade^TM^, Kerr, Orange, CA, USA). According to the manufacturer, this composite matches all 16 VITA classic shades with just the following 3 shades: light, medium, and dark. Table 1 shows the composition of the studied material.

**Figure 1:**
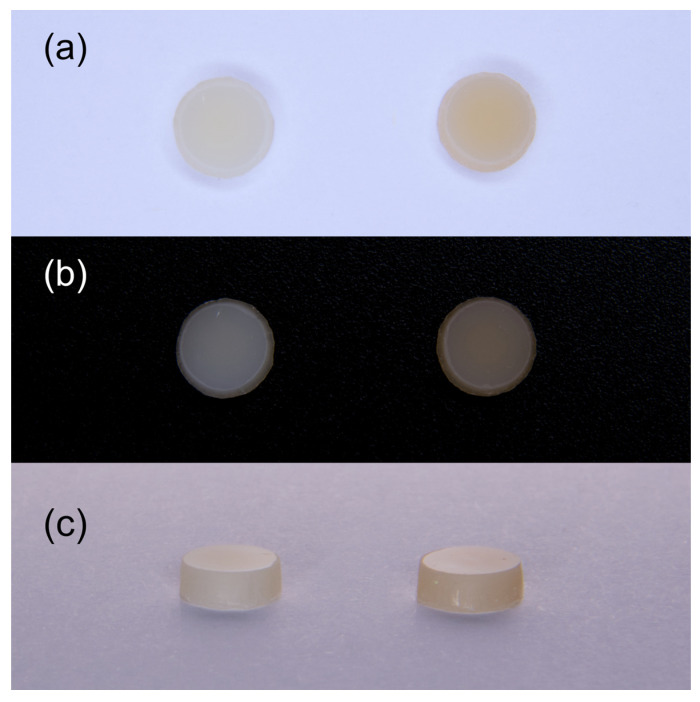
The light (**left**) and dark (**right**) composite specimens against the white (**a**) and black (**b**) backgrounds and their lateral view (**c**).

Sixty composite disks of light (*n* = 30) and dark (*n* = 30) shades were prepared using polytetrafluoroethylene molds (5 mm in diameter and 2 mm thick) (Figure 1). The molds were filled with the tested composite materials and placed between two glass plates covered with a transparent polyester Mylar strip. A load weight was placed on the surface to achieve uniform thickness and extrude the excess material. A polymerization procedure was carried out with the high-power Demi Plus LED light-curing system (from 1100 to 1330 mW/cm^2^) (Kerr). Irradiance was measured using a radiometer (L.E.D. radiometer, Demetron/Kerr, Orange, CA, USA). The specimens were cured at 1 mm distance (thickness of the glass plate) for 10 s (light composite shade) or 20 s (dark composite shade), in accordance with manufacturer’s recommendations. No surface treatments were applied to eliminate possible polishing-related changes [15]. All specimens were examined for surface defects under magnification (10×) with the use of a dental operational microscope (OPMI PROergo, Carl Zeiss Meditec AG, Oberkohen, Germany). Disc thickness was verified using a caliper at three different specimen locations. To complete the polymerization, the specimens were stored in distilled water at 37 °C for 24 h in a dark chamber.

Specimens (30 of each shade) were randomly divided (using a random sequence generated online at randomizer.org) into the following 3 groups (*n* = 10) according to the prophylaxis procedure used: (1) ultrasonic scaling [US], (2) air-powder polishing with sodium bicarbonate [AP], and (3) controls (no surface treatment) [C].

### 2.2. Ultrasonic Scaling

The US specimens were ultrasonically scaled using a SATELEC (Satelec Acteon Group, Bordeaux, France) piezoelectric scaler with an N1 tip under water cooling, for 10 s each, at a low power setting (level 3). The scaler tip was angled at 15–20° to the surface and was moved across the specimen with parallel strokes.

### 2.3. Air-Powder Polishing

The AP specimens were air-polished by PROPHYflex 3 (KaVo, Biberach, Germany) for 10 s each, at 4 bars of pressure at a 60-degree angle and 5.0 mm away from the surfaces. Sodium bicarbonate powder with particle size of 60 μm–70 μm (PROPHYflexTM Powder, Kavo, Biberach, Germany) was used in the study.

To prevent operator variability, all instrumentations were performed by one operator. The specimens were rinsed in running tap water for 30 s, cleaned in an ultrasonic bath for 5 min, and submitted for translucency and color analysis.

### 2.4. Translucency and Color Stability Assessment

A custom-built setup was used to measure reflection spectra in 45/0 configuration and determine color coordinates in the CIELAB color space. Light of a stabilized tungsten-halogen lamp (SLS201L/M, Thorlabs, Newton, NJ, USA) was transmitted through an optic fiber with a core diameter of 600 μm, NA 0.22, and collimated using a microscope objective with a magnification of 10× (f = 16 mm) and projected on the sample surface at an angle of 45 degrees in such a way that the spot size at its widest point was 4 mm. To collect the reflected signal at 0 degrees to sample normal, a second collimator connected to a fiber with a core diameter of 400 μm, NA 0.39, was used. The detection spot with a size of 1 mm was located at the center of the illumination area. The collected signal was detected using an OceanOptics Maya200pro (Ocean Insight, Orlando, FL, USA) spectrometer in the range of 200–1100 nm with a spectral resolution of 10 nm. Each of the samples was measured on black and white background before and after treatment. Diffuse reflectance standards with reflectance of 99.0% and 0.95% in the range of 350–800 nm (LabSphere, North Sutton, NH, USA) were used as substrates. The recorded spectra were smoothed using a moving average with a window of 10 pixels (5 nm).

The reflection spectrum on white and black substrates $R_{s}^{w,b}\left(\lambda \right)$ was calculated as follows:

$$R_{s}^{w,b}\left(\lambda \right)=\frac{I_{s}^{w,b}\left(\lambda \right)-I_{d}\left(\lambda \right)}{I_{l}\left(\lambda \right)-I_{d}\left(\lambda \right)}$$

where $I_{s}^{w,b}\left(\lambda \right)$ is the spectrum of the sample on a white and black background, respectively, $I_{d}\left(\lambda \right)$ is the dark spectrum measured in the absence of light, and $I_{l}\left(\lambda \right)$ is the spectrum measured for a white standard. The white standard was positioned in such a way that its surface was at the level of the samples’ surface.

The reflection spectra of the samples were measured three times to take into account possible inhomogeneity. The coordinate values in the CIEXYZ color space were calculated using the color-science library [37] with CIE 1931 2-degree standard observer color-matching function and standard illuminant D65 for each reflection spectrum. After that, the obtained values were converted into the CIELAB color space and averaged. 

The CIELAB coordinates of the white and black substrates calculated from the reflectance spectra of the calibration certificates were 99.63, −0.028, 0.051 and 8.57, 0.0004, 0.0011, respectively.

The translucency parameter (TP) was then calculated according to the following formula:

$$\mathrm{TP}=\sqrt{{\left(L_{1}^{*}-L_{2}^{*}\right)}^{2}+{\left(a_{1}^{*}-a_{2}^{*}\right)}^{2}+{\left(b_{1}^{*}-b_{2}^{*}\right)}^{2}},$$

where L*_1_, a*_1_, and b*_1_ indicate readings on the white background and L*_2_, a*_2_, and b*_2_ indicate readings on the black background.

After prophylaxis treatment and baseline measurements, all composite discs were immersed in coffee. The coffee was freshly brewed using Brazilia Santos dark-roasted beans and an espresso coffee machine. Boiling water was added to obtain 200 mL of solution, followed by stirring and cooling to room temperature. Each specimen was immersed in separated vials containing 50 mL of coffee solution. After a 48-h staining period, the specimens were gently rinsed and stored in distilled water at 37 °C before final translucency and color measurements.

The total color change (ΔE) was calculated according to the following formula:

$$\Delta E=\sqrt{{\left(L_{1}^{*}-L_{2}^{*}\right)}^{2}+{\left(a_{1}^{*}-a_{2}^{*}\right)}^{2}+{\left(b_{1}^{*}-b_{2}^{*}\right)}^{2}},$$

where L*_1_, a*_1_, and b*_1_ indicate initial readings and L*_2_, a*_2_, and b*_2_ indicate final readings (after storage in coffee).

### 2.5. Statistical Analysis

Data analysis was accomplished using R version 3.6.0 (26 April 2019). The normality of distribution and equality of variances were confirmed using the Shapiro–Wilk test and Levene’s test, respectively. The data were presented as means and standard deviations for each group. A repeated measures mixed ANOVA test was performed followed by a post-hoc Tukey HSD test for independent groups with adjustment for multiple comparisons and a repeated measures *t*-test for dependent groups.

## 3. Results

**Figure 2:**
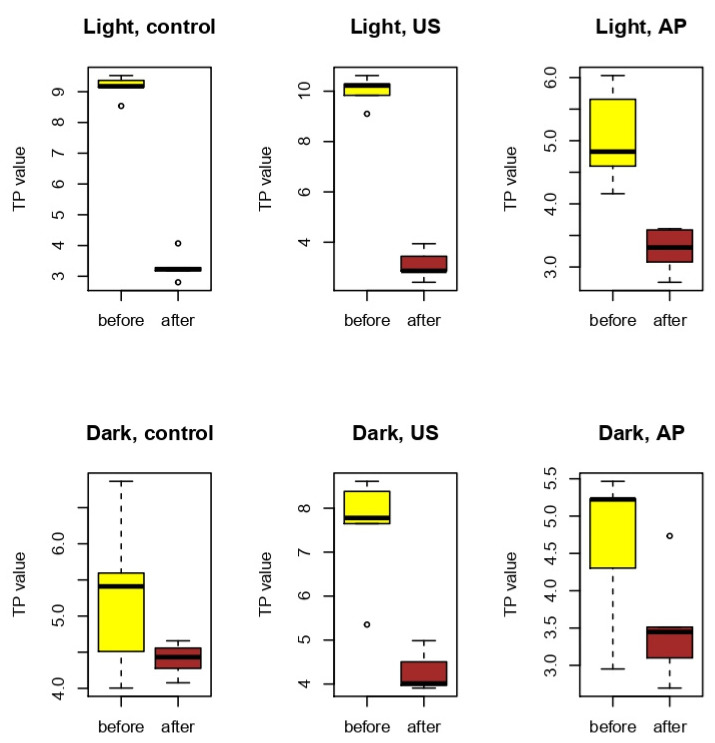
TP values in the studied groups before and after immersion in coffee.

The shade of the composite, method of surface treatment, and interaction of these factors had a significant impact on the translucency of the studied samples at baseline (*p* < 0.01). The initial translucency of the untreated specimens differed significantly between composite shades (*p* < 0.0001). TP values in the light shade and dark shade control groups ranged between 8.54 and 9.53 and 4.00 and 6.86, respectively (Figure 2). There was a significant decrease in TP values after hygienic treatment with air-powder polishing in the light composite group (*p* < 0.0001). Surface treatment with an ultrasonic scaler caused a significant increase in translucency in the dark group (*p* = 0.0073844) but did not alter this parameter in the light group (*p* = 0.06744040). All specimens in the light groups and US specimens in the dark group became significantly more opaque after 48-h immersion in coffee solution. After storage in coffee, only the “shade” factor had a significant impact on translucency values (*p* = 0.000156). No differences were found among different surface treatments within the shade groups (Table 2).

Light shade composite specimens in the C and US groups exhibited significantly greater loss of translucency after coffee immersion compared to dark shade composite specimens (*p* < 0.0001). Translucency changes in the AP groups of both shades were similar (*p* = 0.8746548) (Table 3).

The shade of the composite was the only significant factor for the color change of the studied samples after storage in coffee solution (*p* = 0.0219), mainly due to alterations in the a* parameter (Table 4).

## 4. Discussion

In this study, we assessed the effects of ultrasonic scaling and air-powder polishing on the translucency and color stability of a simplified shade nanohybrid composite. We found that the shade of the composite, method of surface treatment, and interaction of these factors had a significant impact on the translucency of the studied samples at baseline. The light-shade composite specimens showed higher translucency compared to that of dark shade composite specimens.

According to the manufacturer, OptiShade does not require the use of dentin shades due to optimal opacity similar to that of natural tooth tissues. In the present study, the TP values of composite specimens without surface treatment varied from 8.54 to 9.53 in the light group and from 4.00 to 6.86 in the dark group. The values obtained were higher than we expected. Babina et al. showed that TP values of different multishaded composites varied from 1.5 to 3.9 for opaque shades and from 3.9 to 5.4 for most enamel shades. In their study, Ceram X duo E2 was the only shade that demonstrated extremely high translucency (10.91 ± 0.11) [14]. The differences between TP values can be explained by the variability of the experimental protocols [38].

The translucency and color stability may depend on the roughness of the composite surface [39], which, in turn, may be increased during dental prophylaxis [40,41]. It was found that both ultrasonic scaling and air-powder polishing increased composite surface roughness; air-powder polishing with either calcium carbonate or sodium bicarbonate produced a greater increase in surface roughness of composite resin than ultrasonic scaling [35]. In our study, there was a decrease in translucency after air-powder polishing in both groups; however, the differences were significant only for the light composite group. In contrast, ultrasonic scaling caused a slight but significant increase in translucency in the dark group and did not alter this parameter in the light group. However, Colucci et al., who assessed the effect of air-powder abrasion on composite translucency, found that sodium bicarbonate air-powder polishing alone had no effect on material translucency yet increased the changes in translucency associated with staining media [42].

In the present study, we assessed not only the effect of prophylactic surface treatment but also the effect of composite shade on the samples’ translucency. Interestingly, light-shade composite specimens in the C and US groups exhibited a marked loss of translucency after coffee immersion compared to dark-shade composite specimens, while translucency changes in the AP groups were minor and did not differ significantly between the shades.

The storage in coffee solution caused surface staining in all groups as follows: ΔE parameter ranged between 2.69 and 12.05. The shade of the composite was the only significant factor for the color change, mainly due to alterations in the a* parameter (significant) and partially due to alterations in the b* parameter (a tendency). Samples in the light group tended to exhibit greater staining compared to that in the dark group. The type of surface treatment had no effect on the severity of staining. Partially comparable results were reported by Gaglianone et al. who found no effect of air abrasion combined with immersion in the staining media on L* and ΔE parameters. However, the authors observed significant changes in b* (for coffee and red wine) and a* (for red wine) values in comparison with the untreated surfaces [43].

Researchers have no common approach to clinical criteria of color change acceptability. Most researchers believe that ∆E ≤ 1 is not appreciable by the human eye [44,45]. According to some studies, ∆E values > 1 and < 3.3 are clinically acceptable but can be recognized by skilled operators [44]. In this range, ∆E > 1 and < 2 is frequently detected by some observers and ∆E > 2 are detected by all observers [46]. Taking into account the individual features and differences in the structure and morphology of organs and tissues, most researchers consider ∆E > 3.3 [44,45,47] as clinically unacceptable. In our study, coffee caused detectable color changes (∆E > 2) in all tested specimens.

Nowadays, there is a paucity of the literature on translucency and color stability of simplified shade composites. The existing studies vary greatly in their experimental design, and their results are difficult to compare. In a study by Sulaiman et al., a universal chromatic nano-filled composite (8 shades) was considered the most color stable with the least change in its translucency after aging in different media compared with multi-shaded micro-hybrid and nano-hybrid composites. The most pronounced discoloration of all tested materials occurred in the groups immersed in coffee [16]. A study by Gurgan et al. showed satisfactory color stability and relatively high translucency of a 5-shade universal composite [48]. Lucena et al. compared the optical behavior of one-shaded and group-shaded composite materials and found that optical properties differed significantly between the groups. One-shaded composites showed significantly greater translucency values than those of group-shaded material for the same thicknesses [49]. Ebaya et al. reported that the aging procedure negatively affected color stability and surface characteristics of ormocer-based and metacrilate-based single-shade restorative materials [22].

We readily acknowledge several limitations to our study. First, the study did not assess the surface characteristics of the materials. Second, composite specimens were prepared using Mylar strips without finishing and polishing. However, in clinical settings, restorations routinely require final treatment for contouring, occlusal adjustment, and the removal of excess material. Third, we assessed a single composite resin and used only one type of powder (sodium bicarbonate). Besides, extended coffee immersion time could have caused more pronounced changes in composite optical characteristics.

## 5. Conclusions

Within the limitations of this in vitro study, the following conclusions may be drawn: air-powder polishing with sodium bicarbonate powder significantly decreased the translucency of the light shade composite specimens; translucency and color stability of ultrasonically scaled specimens were similar to those of controls; 48-hour immersion in coffee solution caused a pronounced staining in all groups; the shade of the composite was the only significant factor that influenced color changes after storage in coffee, while treatment method had no effect on this parameter. Further research may be aimed at comparing different types of composites, different powders, and different immersion times.

## Figures and Tables

**Table 1:** Composition of the resin composite material used in this study.

Resin Composite	Manufacturer	Filler Type	Filler Loading, % by Weight
OptiShade^TM^	Kerr, Scafati, Italy	Selection of chemically infused mixed oxides, pre-polymerized filler, barium glass filler, silica, and ytterbium trifluoride (40 nm–30 μm)	81.5

**Table 2:** Mean translucency (TP) values (standard deviation) of the tested specimens at baseline and after immersion in coffee solution.

Group	Baseline	Coffee	*p* Value (Before-After)
Light			
	9.15 (0.38) ^a^	3.31 (0.46) ^ACD^	<0.0001
C US AP	10.01 (0.58) ^a^	3.10 (0.60) ^AC^	<0.0001
	5.05 (0.77) ^b^	3.27 (0.36) ^A^	0.00154
Dark			
	5.28 (1.10) ^b^	4.40 (0.23) ^B^	0.119
C US AP	7.55 (1.30) ^c^	4.28 (0.46) ^BD^	0.000703
	4.63 (1.04) ^b^	3.50 (0.76) ^ABC^	0.0845
*p* value (“shade” factor)	<0.0001	0.000156	
*p* value (“treatment” factor)	<0.0001	0.128261	
*p* value (“interaction” factor)	0.00124	0.091941	

a–c and A–D—different letters indicate statistically significant differences among the groups; US—ultrasonic scaling; AP—air-powder polishing with sodium bicarbonate; C—no surface treatment (controls).

**Table 3:** Mean translucency difference (∆TP) values (standard deviation) of the tested specimens at baseline and after immersion in coffee solution.

Group.	C Group	US Group	AP Group
Light	−5.84 (0.82) ^a^	−6.91 (0.58) ^a^	−1.79 (0.81) ^bd^
Dark	−0.88 (1.05) ^b^	−3.28 (1.16) ^d^	−1.14 (1.04) ^b^
*p* value (light-dark)	<0.0001	<0.0001	0.8746548

a–d—different letters indicate statistically significant differences among the groups; US—ultrasonic scaling; AP—air-powder polishing with sodium bicarbonate; C—no surface treatment (controls).

**Table 4:** Mean CIELab color difference values (standard deviation) of the tested specimens at baseline and after immersion in coffee solution.

Group	∆L	∆a	∆b	∆E
Light				
	1.90 (1.06)	−0.14 (0.24) ^a^	8.43 (2.44)	8.75 (2.19) ^a2^
C US AP	2.67 (1.08)	0.12 (0.15) ^a^	6.40 (2.02)	7.11 (1.53) ^a^
	0.50 (2.65)	0.19 (1.15) ^a1^	6.37 (2.94)	7.11 (2.22) ^a^
Dark				
	1.14 (1.37)	−0.75 (0.57) ^a^	5.51 (2.00)	5.91 (1.71) ^a^
C US AP	1.45 (1.09)	−0.27 (0.56) ^a^	6.60 (1.31)	6.87 (1.17) ^a^
	2.14 (2.05)	−1.08 (1.03) ^a1^	3.99 (2.58)	5.36 (1.75) ^a2^
*p* value (“shade” factor)	0.851	0.0084475	0.0517	0.0219
*p* value (“treatment” factor)	0.596	0.43764	0.2107	0.3944
*p* value (“interaction” factor)	0.141	0.38221	0.2811	0.2832

a–d—different letters indicate statistically significant differences among the groups; US—ultrasonic scaling; AP—air-powder polishing with sodium bicarbonate; C—no surface treatment (controls); ^1^—indicates a tendency toward significant differences between groups (∆a between dark AP and light AP groups, *p* = 0.096); ^2^—indicates a tendency toward significant differences between the groups (∆E between the dark AP and light C groups, *p* = 0.06328).

## Data Availability

The data are available from the corresponding author upon reasonable request.
